# First identification of *Strongyloides stercoralis* infection in a pet dog in Argentina, using integrated diagnostic approaches

**DOI:** 10.1186/s13071-023-06022-6

**Published:** 2023-10-27

**Authors:** Pablo Borrás, Matías Gastón Pérez, Silvia Repetto, Juan Pedro Barrera, Marikena Guadalupe Risso, Ana Montoya, Guadalupe Miró, Federico Fernandez, Laura Telesca, Collette Britton, Paula Ruybal

**Affiliations:** 1 Centro de Ciencias Veterinarias, Universidad Maimonides, Buenos Aires, Argentina; 2 School of Biodiversity, One Health and Veterinary Medicine, University of Glasgow, Glasgow, UK; 3 Facultad de Medicina, Departamento de Microbiología, Universidad de Buenos Aires, Buenos Aires, Argentina; 4 Instituto de Investigaciones en Microbiología y Parasitología Médica (IMPaM), CONICET—Universidad de Buenos Aires, Buenos Aires, Argentina; 5 Hospital de Clínicas “José de San Martín”, Universidad de Buenos Aires, División Infectología, Buenos Aires, Argentina; 6 Animal Health Department, Veterinary Faculty, Universidad Complutense de Madrid, Madrid, Spain; 7 Laboratorio INNOLAB, Buenos Aires, Argentina; 8 Private Practice, Veterinaria a Domicilio, Buenos Aires, Argentina

**Keywords:** *Strongyloides stercoralis*, Domestic dog, One Health, Parasite biodiversity, Moxidectin

## Abstract

**Background:**

*Strongyloides stercoralis* is a soil-transmitted intestinal nematode with a complex life cycle that primarily affects humans, non-human primates, dogs, and occasionally cats. This study presents, to the best of our knowledge, the first case of *S. stercoralis* infection and its genotyping in a domestic dog from Argentina.

**Methods:**

The patient was a female wired-haired Teckel dog exhibiting recurrent coughing. Coproparasitological analysis using the Baermann technique revealed the presence of rhabditiform larvae morphologically compatible with *S. stercoralis*. To confirm this finding, molecular diagnosis (18S ribosomal RNA) and analysis of the *cox1* gene were performed.

**Results:**

We identified a haplotype (HP20) that has previously only been related to *S. stercoralis* infection in dogs, but was found in the present study to be highly related to the haplotype (HP16) of a zoonotic variant and divergent from those previously described from human patients in Argentina. Furthermore, unlike in human cases following treatment with ivermectin, the dog was negative after moxidectin treatment according to polymerase chain reaction of the sampled faeces.

**Conclusions:**

This case report shows the importance of further investigation into potential transmission events and prevalences of *S. stercoralis* in dogs and humans in South America. The results reported here should also encourage future work that examines different scenarios of infection with *S. stercoralis* in dogs and humans with the aim of integrating clinical management, diagnosis, treatment and follow-up strategies in the quest for new approaches for the treatment of this disease in animals and humans. The findings support the adoption of a One Health approach, which recognizes the interconnectedness between animal and human health, in addressing parasitic infections such as strongyloidiasis.

**Graphical Abstract:**

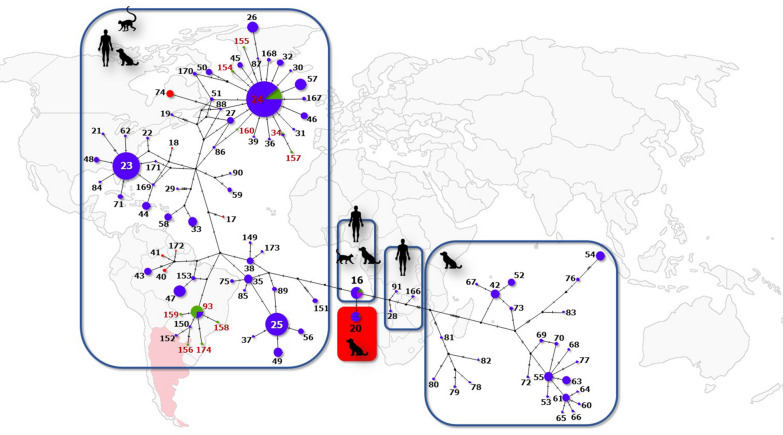

**Supplementary Information:**

The online version contains supplementary material available at 10.1186/s13071-023-06022-6.

## Background

*Strongyloides stercoralis* is a soil-transmitted intestinal nematode that affects humans, non-human primates, dogs, and occasionally cats [1–3] in tropical and subtropical areas around the world [4, 5]. It has also been reported, in both dogs and humans, in temperate climates in countries such as Norway, Poland, Austria and Switzerland [6–8]. Human strongyloidiasis, a neglected tropical disease, is endemic in the northern provinces of Argentina [9–12]. In South America, *S. stercoralis* infections in dogs have been reported in Brazil and Chile, but there have been no clinical or molecular reports of *S. stercoralis* in dogs from Argentina [13].

The life cycle of *S. stercoralis* is complex, involving both parasitic and free-living stages [14, 15]. Rhabditiform first-stage larvae (L1) are released in the faeces of an infected host [16]. These larvae can develop into free-living adult males and females or infective filariform larvae (L3). The L3 can penetrate the skin of a host, migrate to the lungs, and eventually make their way to the small intestine, where they mature into female worms. Also, the L3 can be ingested by the host and develop into female adults in the small intestine. The female adult worms produce eggs by parthenogenesis, which hatch into L1 larvae [17]. The rhabditiform larvae can either be excreted in the faeces or develop into infective filariform larvae within the host’s intestine, which can reinfect the host by penetrating the intestinal mucosa or perianal skin. This ability for autoinfection of the host is unique to *S. stercoralis* and can result in chronic and persistent infections [18]. In dogs, experimental transmammary transmission has also been reported [19], and this form of transmission is likely to occur in natural conditions [20].

Canine strongyloidiasis is typically asymptomatic; however, when the parasitic load is high, gastrointestinal and/or respiratory conditions may occur [21–23]. Dermatological and neurological signs have also been reported [7], which are more frequent in young or immunocompromised dogs [7, 8, 24–26]. In immunocompromised hosts, hyperinfection with parasite migration to other tissues may occur [22]. This has also been associated with the long-term administration of glucocorticoids [7]. Autoinfection in dogs occurs rarely and is associated with immunosuppression [27].

Coproparasitological techniques, such as flotation or sedimentation of faecal samples, show a low sensitivity (4–8%) for the detection of L1 larvae [28, 29]; identification becomes difficult since larvae undergo morphological alterations due to the use of hyperosmotic reagents [30]. The gold standard coproparasitological technique is the Baermann method, which allows the retrieval and identification of L1 larval stages of *S. stercoralis*, and has a sensitivity ranging from 40 to 80% [30]. The technique's sensitivity increases when using samples collected over multiple days [26]. For specific diagnosis of *S. stercoralis* infection, agar plate culture to the L3 stage and molecular methods such as polymerase chain reaction (PCR) can be performed [31, 32].

Fenbendazole and different macrocyclic lactones, such as ivermectin, moxidectin and milbemycin, are used for the treatment of *S. stercoralis* infections in dogs [7, 8, 22–26, 33]. However, no controlled studies have been performed on their use in dogs, which is based only on prior experience or reported clinical cases.

Transmission of this parasite needs to be investigated more thoroughly, to clarify whether infection may be zoonotic or species specific. Previous assumptions regarding the high frequency of zoonotic transmission have been challenged by recent comparative studies, which identified two genetically distinct populations of *S. stercoralis* in dogs [34]. The emergence of different genotypes has sparked discussions about the existence of host-specialized populations and the potential for zoonotic transmission. While one population appears to be dog specific, the other is shared by dogs and humans. These findings are based on analysis of the hypervariable regions I and IV of the nuclear 18S ribosomal RNA (rRNA) small subunit gene and the mitochondrial cytochrome oxidase subunit I (*cox1*) locus [34, 35].

Various screening studies have been performed for soil parasites in urban public spaces in Argentina, employing serological and coproparasitological flotation/sedimentation techniques [36–43]. However, no clinical cases or molecular characterization of *S. stercoralis* have been reported for dogs from Argentina. This study reports, to the best of our knowledge, the first case of *S. stercoralis* in a domestic dog in Argentina, its molecular characterization, clinical management, and phylogenetic analysis. We also describe the patient’s treatment and follow-up, which is of relevance for a One Health intervention approach.

## Methods

### Clinical case

A female wired-haired Teckel dog, 7 months old, from Buenos Aires, Argentina, was taken to the veterinary clinic because of a recurrent cough, which had evolved over a period of 15 days. The dog was acquired from a commercial kennel in Buenos Aires when it was 45 days old. The patient lived with a sibling of the same litter and an adult dog. All the dogs were vaccinated against canine distemper virus, canine adenovirus, canine parvovirus, canine parainfluenza, four leptospirosis serovars and *Bordetella bronchiseptica*. The dogs were routinely dewormed with a combination of pyrantel pamoate, fenbendazole and toltrazuril, and received oral fluralaner every 3 months for the prevention of fleas and ticks. The patient lived in an apartment with no outdoor access and was frequently taken to different urban public spaces and parks. The patient had no history of travel.

Under physical examination a positive cough reflex and mild dry rhonchus were observed. A thoracic X-ray revealed a slight thickening of the bronchial walls compatible with chronic bronchitis. No alterations were found on echocardiogram. Haematological and blood chemical parameters were within the normal ranges (data not shown).

Kennel cough was suspected, so corticosteroid therapy with prednisolone was started at 0.5 mg/kg per os, with the dosage decreased until the medication was stopped after a total of 10 days. The patient showed no clinical improvement, with the frequency of coughing increasing.

### Microscopic examination

The Baermann technique was performed for the detection of rhabditiform larvae (L1) [44]. Briefly, a walnut-size stool sample (3–5 g of faeces) was placed on gauze inserted into a glass funnel which was subsequently filled with warm water up to two-thirds of the sample. The sample was left at room temperature for 24 h. The liquid was collected, and after centrifugation, the sediment was examined under a ZEISS Primostar 3 Digital microscope at ×10 and ×40 magnification for the presence of *S. stercoralis* larvae [45, 46]. An aliquot was stored at – 20 °C for molecular analysis.

### Molecular diagnosis and genotyping

DNA extraction was performed on 250 mg of the faecal sample using DNA PuriPrep-Suelo Kit (Inbio Highway, Argentina) following the manufacturer’s instructions, together with three freeze–thaw cycles in liquid nitrogen during the lysis step. First, a 101-base pair (bp) segment of the conserved region of the *S. stercoralis* 18S rRNA gene was amplified using *S. stercoralis-*specific primers (Stro 18S-1530F 5′-GAATTCCAAGTAAACGTAAGTCATTAGC-3′ and Stro 18S-1630R 5′-TGCCTCTGGATATTGCTCAGTTC-3′) [47]. This primer set has previously been used for the specific detection of *S. stercoralis* in human and dog faecal samples [48, 49]. PCR was performed in a final volume of 15 µL with 0.95 U DreamTaq Hot Start DNA Polymerase (ThermoFisher), 0.1 mg/L bovine serum albumin (BSA; New England Biolabs), 0.2 mM of each deoxynucleoside triphosphate (dNTP) and 0.125 µM of each primer (Inbio Highway), and 1.5 µL of extracted DNA as template. A linearized pZErO plasmid containing a sequence of *Arabidopsis thaliana* was used as a heterologous internal control to verify DNA extraction and integrity [50]. All clinical samples were co-extracted with 200 pg of recombinant plasmid that was amplified in a final volume of 15 μL reaction mixture containing 0.2 μM of each primer (ISFw 5′-AACCGTCATGGAACAGCACGTAC-3′ and ISRv 5′-CTAGAACATTGGCTCCCGCAACA-3′), 0.4 mM of each dNTP (Inbio Highway), PCR buffer at 1 × final concentration (inhibitor-resistant buffer provided with the enzyme; Inbio Highway), 0.1 μg/μL of BSA (New England Biolabs), 2 mM of MgCl_2_, 1.25 U of T-Plus polymerase (Inbio Highway). Cycling conditions for both the 18S rRNA gene fragment and pZErO plasmid were as follows: 3 min at 95 °C, 35 cycles of 45 s at 95 °C, 1 min at 55 °C, and 45 s at 72 °C, with a final elongation step of 5 min at 72 °C. Nuclease-free water and stool DNA from healthy subjects were used as negative controls. DNA isolated from *S. stercoralis* larvae obtained from human positive cultures was used as the positive control [31].

For amplification of the *cox1* gene, primers of Zhou et al. [51] were used, as follows: ZS6985 (5′-GGTGGTTTTGGTAATTGAATG-′3) and ZS6986 (5′-ACCAGTYAAACCACCAATAGTAA-′3). PCR was performed in a 25-μL reaction mixture containing 0.2 μM of each primer, 0.4 mM of each dNTP (Inbio Highway), PCR buffer at 1 × final concentration (inhibitor-resistant buffer provided with the enzyme, Inbio Highway), 0.1 μg/μL of BSA (New England Biolabs), 2 mM of MgCl_2_, 1.25 U of T-Plus polymerase (Inbio Highway) and 2 μL of purified DNA as template. Cycling conditions included an initial 2-min denaturation step at 94 °C, followed by 35 amplification cycles (denaturation at 94 °C for 30 s, annealing at 47 °C for 15 s and elongation at 72 °C for 90 s) and a final extension step at 72 °C for 5 min.

PCR amplifications (18S rRNA and *cox1*) were carried out in an Esco Swift Maxi Thermal Cycler Block (Esco, MO). Seven microlitres of each amplified product was electrophoresed in a 3% agarose gel stained with GelRed Nucleic Acid Gel Stain (Biotium, CA) together with a molecular size marker (MassRuler Express Forward DNA Ladder Mix; ThermoFisher Scientific, MA) to confirm PCR product size and visualized in an Automated BioSpectrum Imaging System (Analytik Jena).

For species confirmation and genotyping analysis, *cox1* PCR products (837 bp) were directly sequenced by Macrogen (Seoul, South Korea). Consensus sequences were obtained through forward and reverse strands assembly using Staden Package software (MRC-LMB, UK). We also visually checked the sequence trace of both strands for the detection of ambiguous sites when two peaks overlapped in both chromatograms. Consensus sequences were aligned and trimmed in frame (404 bp) using MEGA11 software [52]. The obtained sequence was deposited in the GenBank database under accession number OQ921397 and compared to a collection of *S. stercoralis cox1* haplotypes previously analyzed by Repetto et al. [53] and recently published [2, 8, 54] (Additional file 2: Table S1). Genealogical associations of the worldwide dataset or American intraspecific genetic diversity of *S. stercoralis* were studied by haplotype network inferred by the median-joining algorithm (epsilon = 0) using PopART software [55].

## Results

### Identification of *S. stercoralis* by the Baermann technique

**Fig. 1:**
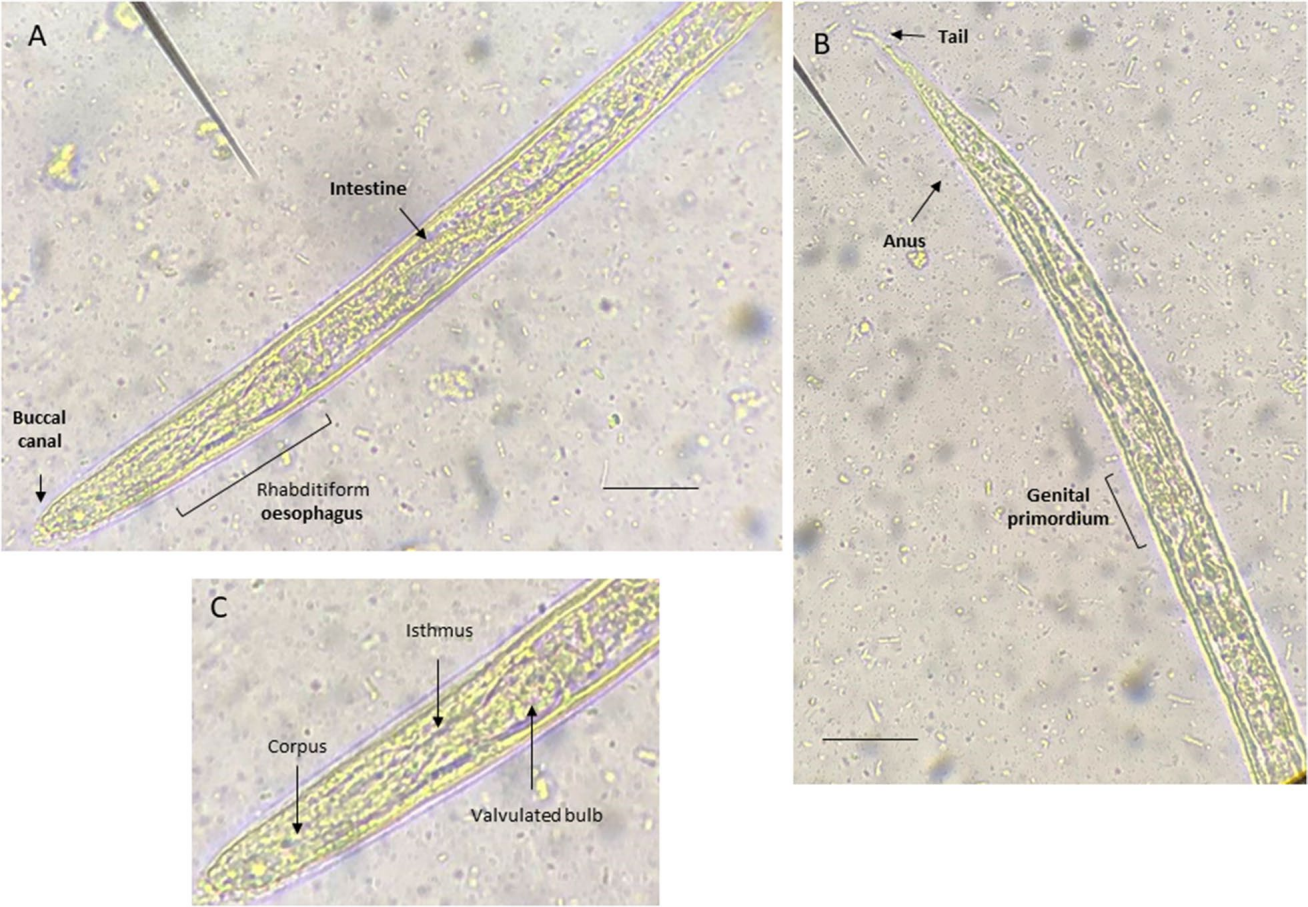
**A**–**C** First-stage (L1) rhabditoid larvae of *Strongyloides stercoralis* isolated from the faeces of a pet dog by the Baermann technique. **A** Detail of the anterior section of the L1 including the buccal canal, oesophagus and intestine. **B** Detail of the posterior end of the L1 including the genital primordium and anus. **C** Characteristics of the oesophagus including the corpus, isthmus and valvulated bulb. Scale bars correspond to 15 μm

The Baermann technique revealed a high number of larvae with a cylindrical body, a tapered anterior end with a buccal cavity, and a pointed, straight tail. The body was covered with a thin cuticle, and there was a prominent longitudinal ridge along the body surface. The corpus, isthmus and valvulated bulb were observed in the oesophagus. The length of the larvae was between 160 and 350 μm. These features helped in the morphological identification of L1 of *S. stercoralis* (Fig. 1A–C), and to differentiate them from larvae of *Oslerus osleri*, *Angiostrongylus vasorum*, and *Filaroides* species [56].

### Molecular diagnosis and typing of* S. stercoralis*

**Fig. 2:**
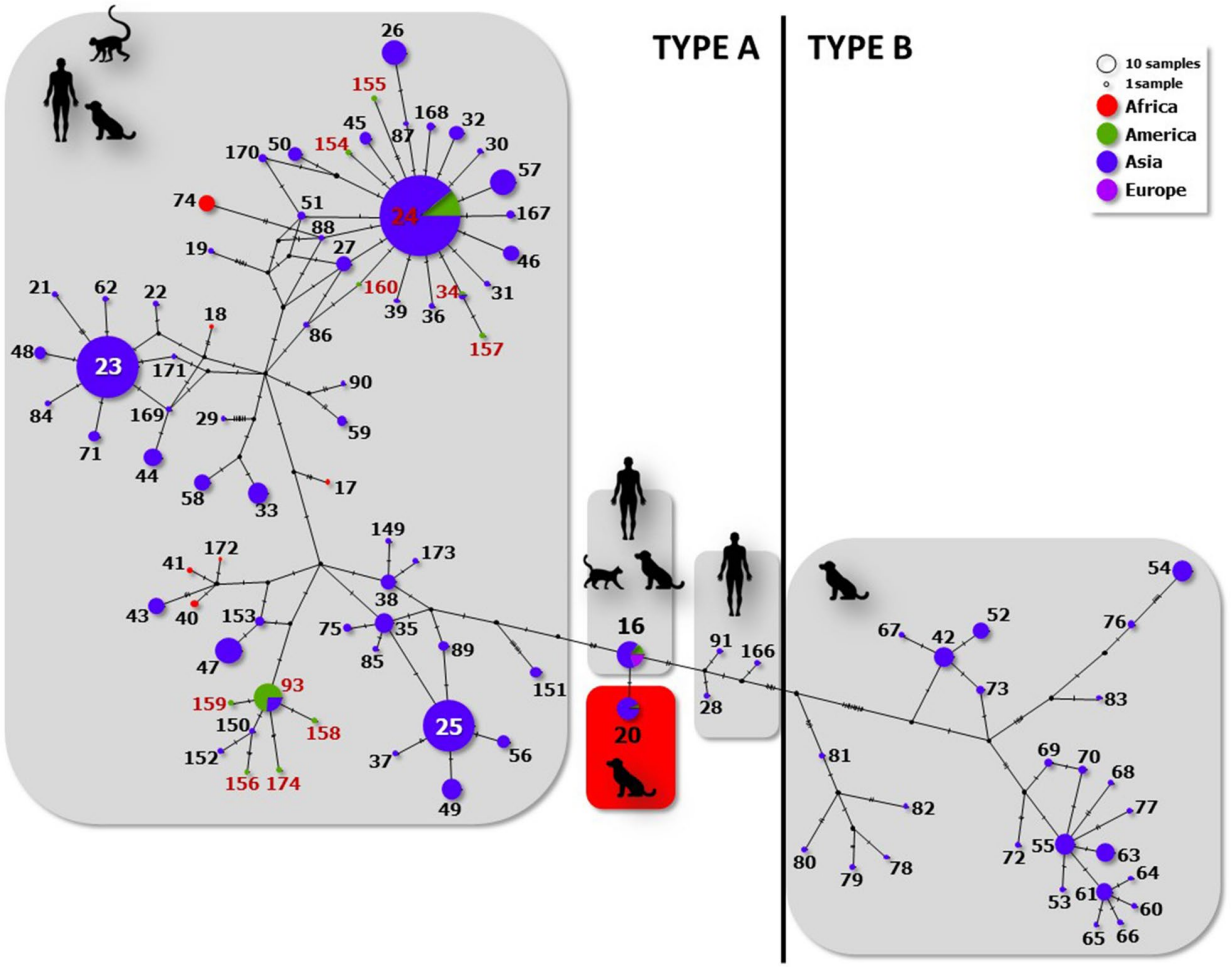
Median-joining network based on a worldwide *Strongyloides stercoralis* haplotype dataset (Additional file 2: Table S1). The nodes are coloured according to the continental distribution [Asia (*blue*), Africa (*red*), Europe (*purple*), America (*green*)].* Grey boxes* indicate cluster organization according to host distribution, while the* red box* highlights haplotype 20 (HP20) (present in the positive case in this study). The numbers in* red* indicate haplotypes previously found in human patients from Argentina

Amplification of a 101-bp fragment of the 18S rRNA gene [47–49] suggested a PCR amplicon product compatible with *S. stercoralis*, in accordance with the internal positive control (Additional file 1: Figure S1). To improve our understanding of *Strongyloides* diversity, PCR amplification and sequencing of the *cox1* gene were performed (accession number OQ921397) [51, 53]. We previously showed [53] that this 404-bp region of the *cox1* is congruent with the taxonomy of the genus *Strongyloides* according to phylogenetic analysis. There is evidence of genetic diversity within *S. stercoralis*, with at least two genetic lineages identified worldwide (Fig. 2). These lineages differ in their geographic distribution, host specificity, and potential zoonotic transmission. So far, type A lineage has a worldwide distribution and is found in humans, dogs, cats, and non-human primates. On the other hand, type B lineage has only been described from dog samples from Myanmar, an hyperendemic region in Southeast Asia [34, 35].

The obtained haplotype in this case corresponded to HP20, according to a previously published haplotype code [53] (Additional file 2: Table S1). As shown in Fig. 2, this variant is closely related to HP16 and distant from haplotypes previously described for human patients in Argentina (HP24, HP34, HP93, HP154-160). Based on the location of HP20 in the haplotype network, this variant has only one mutation that distinguishes it from HP16, a haplotype with a reasonably high prevalence and one of the widest geographic (Asia, Americas, And Europe) and host (cats, dogs, and humans) distributions in the database (Additional file 2: Table S1) [53]. Molecular diagnostic tests were performed on faecal samples from the other dogs that lived in the same home as the patient, all of which were negative.

### Study of human contact

In order to evaluate the possible zoonotic transmission of the parasite, at the time of the pet’s diagnosis and 4 months later, the owner of the dog was asked if they would give a blood sample to determine their eosinophil count, and a fresh faecal sample for microscopic examination, analysis by sedimentation techniques, agar plate culture, and molecular diagnosis using 18S rRNA primers. The pet owner was asymptomatic. They were found to have no eosinophilia (210 cells/mm^3^) at the time of the pet’s diagnosis or 4 months later, when their eosinophil count was 234 cells/mm^3^. *Strongyloides stercoralis* was not detected in the owner’s faecal samples by microscopic examination, agar plate culture or 18S rRNA PCR.

### Treatment and post-treatment evolution

Upon the detection of larvae identified as *S. stercoralis*, the dog was treated with moxidectin (Moxidex, Mayors) at an oral dose of 0.2 mg/kg once per month for 3 consecutive months.

Faecal samples of the two cohabitant dogs were subjected to coproparasitological analysis using the Baermann technique and PCR, and both were negative. However, given the significance of the findings in the patient, the cohabitant dogs were also treated with the same dose of moxidectin as a preventive measure since they shared the same living space and visited the same public spaces as the former.

The patient showed clinical improvement 7 days after the first moxidectin dose, and after 21 days of treatment, the clinical respiratory symptoms had disappeared. Thirty days after the first dose of moxidectin, coproparasitological analysis was performed using the Baermann and PCR techniques. Both the patient and cohabitant dogs were negative for both techniques after treatment. The thoracic X-ray taken 45 days after the first moxidectin dose had been administered revealed an improvement in the patient’s bronchitis. Upon completion of the treatment, the thoracic X-ray showed no signs of abnormality with respect to the pulmonary fields, cardiac silhouette, mediastinal space or pleural cavity.

Four months after the administration of the first dose of moxidectin, faecal samples of the infected dog and the cohabitant dogs were subjected to another coproparasitological analysis using the Baermann and PCR techniques, and the results were negative for both techniques.

## Discussion

To the best of our knowledge, this is the first report of *S. stercoralis* infection and genotyping in a pet dog from Argentina, and the first record of *cox1* molecular characterization in a dog from South America. Most dogs infected by parasites do not exhibit clinical signs; however, several issues can arise due to *S*. *stercoralis* infection. These most commonly include intestinal and/or respiratory disorders [7, 8, 22, 25, 26] and less commonly dermatological and neurological problems [7, 26]. Bronchitis and chronic cough have been more generally reported in young or immunosuppressed dogs [7, 25, 26].

The patient was 7 months old when the respiratory clinical signs appeared, and the administration of corticosteroids worsened the clinical symptoms. Corticosteroids or immunosuppressive drugs may lead to acceleration of the *S. stercoralis* autoinfection cycle, facilitating hyperinfection and spread of the parasite [27, 57, 58]. Unlike strongyloidiasis in humans [59], the dog did not exhibit eosinophilia, which is consistent with other studies that indicated that eosinophilia is not always observed in canines [22, 26]. The Baermann technique is the gold standard for the diagnosis of *S. stercoralis* in dogs. This technique is also used to detect other lungworms that display positive hydro-/thermotropism. To ensure accurate diagnoses, it is important to use taxonomic keys to identify the larvae. It should be noted that faecal samples may also contain larvae of other lungworms, such as *Filaroides* spp. and *Oslerus osleri*, as well as *Angiostrongylus vasorum* and *Crenosoma vulpis*, which need to be differentiated [56]. To avoid contamination with free-living larvae, samples should be collected from the ground as soon as they emerge from the rectal ampulla [5]. It is possible that *S. stercoralis* is under-diagnosed in South America as a consequence of a low index of clinical suspicion for lungworms there in addition to infrequent use of the Baermann technique.

The source of infection was not determined in the present study. The dog had been acquired from a kennel in Buenos Aires, which had been importing breeding dogs from different European countries in recent years. The importation of dogs has been associated with the emergence of clinical cases of* S. stercoralis* in different parts of Europe [7, 8, 26, 60]. Thus the importation of dogs may be a risk factor for introducing this parasite into non-endemic areas [26], and it cannot be ruled out that the infection may have originated in the kennel. The other possible mode of transmission is transmammary transmission, which has been shown to occur both experimentally and naturally [19, 20]. However, this seems unlikely since the sibling of the same litter was negative according to the molecular diagnostic method following extraction with the Baermann technique. The patient frequently visited public parks where it was in contact with other dogs, but *S. stercoralis* has not, as yet, been found in parks or public spaces in Buenos Aires or on the outskirts of the city [37, 39–41]. However, in these studies samples were examined from different parks and squares in Argentina by using conventional coproparasitological techniques such as flotation and sedimentation. These techniques show very low sensitivity for the detection of *S. stercoralis* larvae [28–30], and even if these larvae were present, their morphology is altered in saturated solutions and they become unrecognizable as a consequence [24]. Notably, in chronic infections, the elimination rate of larvae to the environment may be low, or such elimination may happen only intermittently during and after treatment [61, 62]. Use of the Baermann technique rather than the less-sensitive coproparasitological techniques is advisable, and the Baermann technique followed by PCR is associated with a higher sensitivity than the Baermann technique alone [22, 23].

There have been reports of zoonotic transmission of lineages of *S. stercoralis* from dogs to humans, particularly in areas where canine and human populations live in proximity [3, 13]. In the current study, we identified HP20 of the mitochondrial *cox1* gene, a variant that has previously only been described from dogs in Asia [53]. In addition, HP20 is genetically distant from the haplotypes seen in the population of human patients from the same geographical region as the dog in the present study [53]. The distance between these haplotypes may be a result of the introduction of haplotypes with infected animals imported from abroad for breeding purposes, as mentioned earlier, or processes of parasite diversification in the geographical region of the present study.

This work represents a first step in the study of *S. stercoralis* variants in dogs outside areas that are endemic for human strongyloidiasis in Argentina. To the best of our knowledge, HP20 has only been previously found in dogs in Japan, strikingly a region that is also considered non-endemic, and together with HP16 represents a link between the cluster with zoonotic potential (type A) and the hypothetically ancestral and non-zoonotic cluster (type B) [35]. The results of the present study indicate the need to investigate the dynamics of these parasites in different geographic areas and hosts (including non-human primates, domestic animals, etc.) to understand the mechanisms driving cryptic species divergence and how their diversity could impact clinical disease, treatment, and management of their infected hosts [63].

Several different antihelminthic drugs have been reported for the treatment of *S. stercoralis* in dogs—fenbendazole [22, 25, 26, 33], ivermectin [7, 8, 23–25], moxidectin [8, 22, 26] and milbemycin oxime [26]—with variable schemes and results, with fenbendazole showing the most contradictory results [22, 25, 26, 33]. Although the use of ivermectin has been effective in the treatment of dogs [7, 8, 26], it is always prescribed off-label, and poses a risk for dogs with a mutation at the *MDR1* gene [64]. We decided to treat the infected dog with moxidectin since it is approved for use in dogs, is commercially available, and we have prior experience of successful results with its use [8, 22]. It has even been proposed as a treatment for human strongyloidiasis*,* where the drug of choice is ivermectin [65]. However, to the best of our knowledge, no controlled studies on the efficacy of moxidectin for *S. stercoralis* in dogs have been performed. The administration of three consecutive monthly doses was designed to ensure eradication of the parasitosis and to prevent larval contamination of the environment [8].

Repetto et al. [66] considered that parasitological cure of strongyloidiasis in humans after ivermectin administration is unlikely, and found that PCR tests remained positive years after treatment. However, in the present study on a dog, the animal became PCR negative after treatment with moxidectin. This may have been due to three factors that differ between the present study and Repetto et al.’s [66]: the host (a dog rather than humans), the drug used (moxidectin rather than ivermectin), and a divergent parasite genotype. Further research is needed to explore these findings, and specifically, if negative PCR results can be achieved in human patients following alternative therapeutic approaches [66]. In addition, more controlled trials are necessary to evaluate the most effective antiparasitic drugs and the most appropriate treatment schemes for a One Health approach.

## Conclusions

To the best of our knowledge, this is the first report of *S. stercoralis* in a pet dog from Argentina. Veterinarians should include this parasitosis in their differential diagnosis, particularly for young dogs with gastrointestinal and/or pulmonary symptoms. The use of the Baermann technique in these cases will help veterinarians to make a correct diagnosis. The treatment of *S. stercoralis *is challenging, and different antiparasitic drugs have been proposed for this in the literature. More studies are required to elucidate the best therapeutic option for cases of strongyloidiasis, and clinical and parasitological monitoring in these patients is essential.

### Supplementary Information

**Additional file 1: Figure S1. **Molecular detection of *Strongyloides stercoralis* from the faeces of the pet dog.* 1–3* 18S rRNA PCR amplification, 101 base pairs (bp) (*black arrow)*.* 4–6* Internal amplification control (linearized pZErO plasmid containing a sequence of *Arabidopsis thaliana*), 195 bp (*white arrow*).* 1–4* DNA from the faecal sample of the pet dog;* 2* DNA from *S. stercoralis* larvae (18S rRNA positive control);* 5* linearized pZErO plasmid (internal amplification positive control);* 3*,* 6* negative control (water);* 7* MassRuler Express Forward DNA Ladder Mix (ThermoFisher Scientific, MA).

**Additional file 2: Table S1**. Dataset of strains included in the haplotype analysis. Haplotypes were coded based in the diversity of cox1 marker from 1018 strains and according to previously published study [53]. Information includes geographical distribution and year of each clinical case, host category, Strongyloides species and genbank accession number (ACCN).

## Data Availability

All data generated or analyzed during this study are included in this published article.
