# Supplementary figures and images for: First identification of Strongyloides stercoralis infection in a pet dog in Argentina, using integrated diagnostic approaches
Source: Parasit Vectors. 2023 Oct 27;16:389. doi: 10.1186/s13071-023-06022-6 (PMC10605978; doi:10.1186/s13071-023-06022-6)

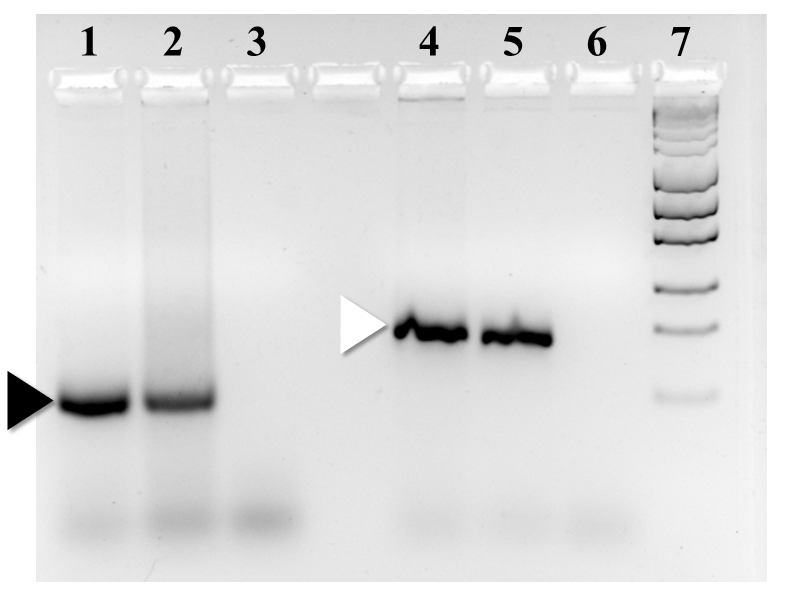

Supplement: Supplementary file 1 — Additional file 1: Figure S1. Molecular detection of Strongyloides stercoralis from the faeces of the pet dog. 1–3 18S rRNA PCR amplification, 101 base pairs (bp) (black arrow). 4–6 Internal amplification control (linearized pZErO plasmid containing a sequence of Arabidopsis thaliana), 195 bp (white arrow). 1–4 DNA from the faecal sample of the pet dog; 2 DNA from S. stercoralis larvae (18S rRNA positive control); 5 linearized pZErO plasmid (internal amplification positive control); 3, 6 negative control (water); 7 MassRuler Express Forward DNA Ladder Mix (ThermoFisher Scientific, MA). [file 13071_2023_6022_MOESM1_ESM.tif]
